# HIV Virologic Failure among Patients with Persistent Low-Level Viremia in Nairobi, Kenya: It Is Time to Review the >1000 Virologic Failure Threshold

**DOI:** 10.1155/2023/8961372

**Published:** 2023-04-27

**Authors:** Mirriam M. Nzivo, Cecilia N. Waruhiu, James M. Kang'ethe, Nancy L. M. Budambula

**Affiliations:** ^1^School of Biological Sciences, Jomo Kenyatta University of Agriculture and Technology, P.O. Box 62000-00200, Nairobi, Kenya; ^2^The Africa Genomics Centre and Consultancy Ltd., P.O. Box 381-00517, Nairobi, Kenya; ^3^Comprehensive Care Centre, Kenyatta National Hospital, P.O. Box 20723-00202, Nairobi, Kenya; ^4^Department of Biological Sciences, University of Embu, P.O. Box 6-60100, Embu, Kenya

## Abstract

Persistent low-level viremia (PLLV) of 200-999 copies/ml has been reported as a risk factor for HIV virologic failure (VF). This retrospective study was aimed at characterizing patients with PLLV, determining factors associated with VF, and determining the effect of regimen change. Data were extracted from electronic medical records for HIV care and treatment. Patients' characteristics (*N* = 705) were as follows: a mean age of 42 years, majority female (55%), and 51% married. A majority (78.7%) had a history of opportunistic infections in their ART lifetime. To determine factors associated with VF, 187 records on patients who maintained PLLV and 12 on deceased patients at the time of data review were eliminated from the analysis, leaving 506 patient records. Out of the 506, 89% (451/506) suppressed VL to nondetectable levels while 11% (55/506) had VF, and the difference was significant (*P* = 0.0001). Virologic failure was significantly associated with ages 10-30 years (*P* < 0.05). Baseline VL ≥ 1000 (OR 3.929; *P* = 0.002) and 200-999 copies/ml (OR 4.062; *P* = 0.004) were associated with VF. During PLLV, factors associated with VF included the following: PLLV of 200-999 copies/ml (*P* < 0.05), viral blips (OR 4.545; *P* = 0.0001), mean maximum VL (*P* < 0.05), and age (*P* = 0.043). Married marital status was inversely associated with VF (OR 0.318; *P* = 0.026). Regimen change was not significantly associated with virologic outcomes. However, patients who switched regimens to the second line had a high risk of VF (*P* = 0.028; OR 3.203). Regimen change was significantly high (*P* < 0.05) among adolescents and patients with a start regimen of 2NRTI+1NNRTI. Most of the PLLV patients (89%) achieved nondetectable VL after their continued ART monitoring for at least 12 months. Therefore, PLLV was not an indicator of VF. However, a consistent VL of ≥200-999 copies/ml at baseline and more than 12 months of ART care and treatment were significantly associated with VF. Patients with VL 200-999 copies/ml, adolescents, and young adults require intensive ART monitoring and support.

## 1. Introduction

The aim of combined antiretroviral therapy (cART) is to hinder viral replication and hence suppress HIV-1 RNA viral load (VL) in the blood to below limits of assay detection. Most of the ART care and treatment guidelines globally have indicated VL as a predictor for monitoring virological response to cART. Unfortunately, some patients on cART still experience detectable VL in their blood in the form of viral blips or persistent low-level viremia (PLLV). According to the World Health Organization (WHO), PLLV is defined as viral loads between 50 and 1000 copies/ml on at least two consecutive measures in patients taking ART. PLLV is estimated to be experienced by 0.4-38.7% of HIV-1-infected people on ART [1, 2]. PLLV has clinical significance as it can lead to antiretroviral drug resistance, virologic failure (VF), immune activation, chronic inflammation, viral evolution, HIV-1 pool expansion, and shedding of the virus, which can result in HIV-1 transmission, AIDS-defining illnesses, and even mortality [3–7].

Some studies have reported PLLV as an independent indicator of VF while others disagree, especially about the significance of low-level low-range viremia. Notably, clinical implications differ among patients with PLLV ≤ 199 copies/ml and patients with higher viremia 200-<1000 copies/ml, with the latter being associated with VF [4, 5, 8–11]. The increasing evidence on the negative impacts of PLLV, especially persistent VL between 20 and 999, has led some organizations in resource-rich settings (RRS) to adopt guidelines lowering the ≥1000-copies/ml threshold of VF set by WHO. For instance, the European AIDS Clinical Society 2020 Guidelines define VF as viral loads ≥ 50 copies/ml [12, 13]. The United States Department of Health and Human Services defines VF as detectable viremia ≥ 200. In Kenya, the National AIDS and STD Control Programme (NASCOP) defines PLLV as having a detectable VL above the lower detection limit value but <1000 copies/ml on two or more consecutive measures and VF as viral loads ≥ 1000 copies/ml [14]. HIV programs in different regions using the WHO guidelines consider patients with VL < 1000 copies/ml as virologically suppressed. There are therefore no standard guidelines on the management of patients with PLLV.

Virologic failure among patients with PLLV can be attributed to the emergence of drug-resistant mutations [1, 7, 14]. For instance, the prevalence of drug resistance at PLLV (50-1000 copies/ml) has been reported at 70-80% in resource-limited settings (RLS) and 10-20% in RRS in several studies [1, 15, 16]. Swenson et al. [2] reported that among 1965 patients with PLLV ≤ 1000, 30% had drug-resistant mutations. Some studies have reported that regimen switch during PLLV can result in suppression of viremia to below detectable levels [1, 6, 17]. A study in Lesotho reported that a switch from a first-line to a second-line regimen resulted in suppression of VL to <50 copies/ml among patients who had persistent viremia between 100 and 999 copies/ml [18]. In addition, Boillat-Blanco et al. [1] reported that patients taking a first-line nonnucleoside reverse transcriptase inhibitor- (NNRTI-) based regimen benefited if they switched regimens. However, some studies have reported that viral outcomes among patients with PLLV were not influenced by regimen change [6, 19, 20]. A large observational cohort study of 70930 participants among patients with LLV between 51 and 999 copies/ml reported that 25% of patients achieved viral suppression below 50 copies/ml without regimen change [6].

There are many studies on PLLV, but most of them have been done in RRS. There is a paucity of information on HIV-1 PLLV in sub-Saharan Africa (SSA), where a high burden of HIV/AIDS is reported. There is therefore a need for studies on PLLV to give information on trends of PLLV, mitigate risks of VF, and help policymakers develop guidelines for managing such patients, especially in SSA. This retrospective study was aimed at characterizing patients with PLLV, determining factors associated with VF among patients with PLLV, and determining the effect of regimen change on virologic outcomes. This study is among the few studies in SSA focusing on PLLV among HIV patients.

## 2. Methods

### 2.1. Study Design and Population

This was a retrospective study from January 2015 to December 2021. Records of HIV-infected patients enrolled in care at the Comprehensive Care Centre (CCC) at Kenyatta National Hospital (KNH) who experienced PLLV during the period were examined. Kenyatta National Hospital is the largest teaching and referral hospital in Kenya and is located in Kenya's capital city, Nairobi. The CCC at KNH has approximately 10000 HIV patients enrolled for care. Study inclusion criteria are as follows: all age groups, all genders, at least two VL measurements 20-999 copies/ml taken six or more months apart, and patients on cART.

### 2.2. Ethical Approval

Approval to carry out this study was granted by the Kenyatta National Hospital/University of Nairobi Ethical Review Committee (ERC). The study ERC number is P234/04/2021. A waiver was granted for obtaining individual informed consent as the research involved no more than minimal risk.

### 2.3. Data Extraction

Data were extracted from the electronic medical record (EMR) system for HIV care and treatment using a data extraction built-in tool and exported to Excel. All patient identifiers such as names, home addresses, and telephone number(s) were removed while retaining only the patient serial numbers. Demographic data abstracted included the age of the patients, gender, level of education, and marital status. Medical history data included the age at HIV diagnosis, WHO stage, number of years one had been HIV positive, history of opportunistic infections (OIs) and noncommunicable diseases, Isoniazid Preventive Therapy (IPT) status, duration on ART, and start ART regimen as well as baseline VL measurement. In addition, the regimen switch, minimum and maximum VL measurements at PLLV, duration of PLLV, levels of the first VL measurement at PLLV, and viral load status at PLLV (persistently ≤199 or ≥200-999 copies/ml or viral blips) were abstracted.

### 2.4. Data Analysis

Data were exported to SPSS v. 21 for analysis. Mean and interquartile ranges for continuous variables (age, duration on ART, minimum and maximum VL, etc.) were determined. For categorical variables (age group, gender, baseline WHO stage, start regimen, OI history, etc.), frequencies were determined. To evaluate the correlates of the outcomes (VF and nondetectable VL), a chi-squared test of association was used. Risk factors for VF were determined using logistic regression. The significance level for all statistical tests was set at 5% (95% CI).

### 2.5. Definition of Terms

Depending on the region, there are numerous definitions of PLLV, VF, and viral blips, as seen in the published literature. It is therefore necessary to define the terms as used in this study. The terms are defined in Table 1.

## 3. Results

**Figure 1:**
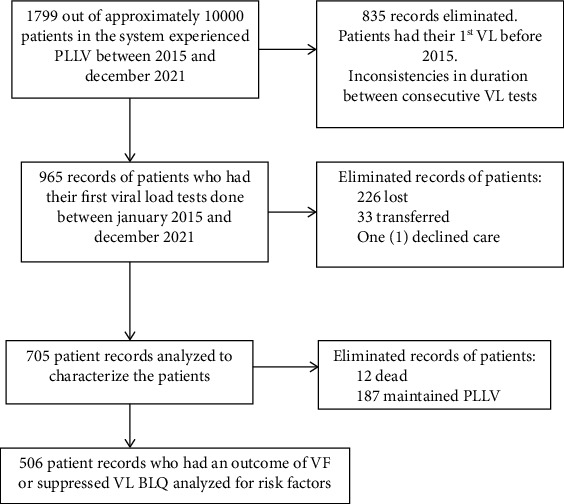
Flow chart of inclusion and exclusion criteria.

A total of 1799 (18%) patients out of approximately 10000 patients in the EMR system at the hospital experienced PLLV between 2015 and 2021. This number was revised to 705 patients based on our inclusion criteria. Patients with baseline VL tests before 2015 were eliminated from the study due to inconsistencies in VL tests done before 2015, when VL was recommended for monitoring disease progression. The inclusion and exclusion criteria have been summarized in Figure 1.

### 3.1. Baseline Characteristics

The patients' mean age was 42 years ± SD 15.1, the HIV diagnosis mean age was 29 years ± SD 15.9, the mean start VL at PLLV was 154 copies/ml ± 192, the mean duration on ART in months was 96 ± SD 43, the mean minimum VL at PLLV was 68 ± SD 99 and maximum 238 copies/ml ± SD 247, and the mean number of months from initiation of ART to PLLV was 44 ± SD 43. Among those who did not maintain PLLV, the mean duration of PLLV to the outcome was 21.6 months ± 8.6. Overall, 34% (240/705) of patients were aged 41-50 years, 55% (388/705) were female, 50.8% (358/705) were married, and 34.6% (244/705) were on WHO stage 1 at the start of ART. Approximately 40% (281/705) of the participants had baseline viral loads of 20-999 copies/ml. The first VL at PLLV for most patients (75.5%, 532/705) was 20-199 copies/ml. It was observed that 19% (133/705) of the patients were diagnosed with HIV at ages 30-39 years and had been positive for more than 10 years (27.5%, 194/705). Patients with a history of opportunistic infections were 78.7% (555/705); the specific OIs have been summarized in Table 2. Overall, 77.3% of the patients had 2NRTI+1NNRTI as their start regimen. More than half (55.3%) had not switched their initial regimen during PLLV (Table 2).

### 3.2. Comparison of Patients Who Became Virally Suppressed and with Virologic Failure

In the analysis of PLLV outcomes, records of patients who had died [12] and those who were still at PLLV (187) were eliminated. Out of the remaining 506 patient records, 451 (89%) had an outcome of nondetectable levels while 55 (11%) experienced VF after the PLLV experience, and this difference was significant (*P* = 0.0001). The factors that significantly affected the outcomes included the following: age (*P* = 0.006), age at HIV diagnosis (*P* = 0.002), marital status (*P* = 0.026), first viral load (*P* = 0.0001), VL status at PLLV (either patients had consistent VL < 200 or ≥200-999 or they had viral blips) (*P* = 0.001), and first VL measurement at PLLV (*P* = 0.0001). None of the selected opportunistic infections and noncommunicable diseases evaluated significantly affected the outcomes (Table 3).

### 3.3. Factors Associated with Virologic Failure

The risk factors for VF in this population were age in general, certain age groups, viral blips, high baseline viral loads, and high VL at the beginning of PLLV, as summarized in Table 4. Married status was inversely associated with VF.

### 3.4. Regimen Switch and Virologic Failure

In this study, 39.7% (201/506) of patients switched regimens at PLLV. Univariate logistic regression showed that regimen switch was not significantly associated with VF (*P* = 0.157) (Table 2). Regimen change was significantly high among patients who had their start regimen as 2NRTI+1NNRTI (OR 8.684; *P* = 0.039). In addition, 80% (161/201) of patients switched to a different first-line regimen while 20% (40/201) switched to second-line regimens, and the difference was significant (*P* = 0.0001). Patients who switched to second-line regimens were more likely to have an outcome of virologic failure (*P* = 0.028) (OR 3.203; 95% CI, 1.13-9.03) than those who switched to a different first-line regimen. Adolescents significantly switched regimens more than the other age groups (OR 2.18; *P* = 0.035).

## 4. Discussion

Approximately 18% of patients attending the CCC at KNH between 2015 and 2021 experienced PLLV. This number falls within the estimated prevalence (0.4-38.7%) of patients who experience PLLV while on ART [1, 9]. A large observational cohort study in South Africa reported that LLV occurred in 23% of HIV patients [6]. The proportion observed in our study indicates that patients in this population could be experiencing several factors associated with PLLV during treatment, such as microbial translocation, inflammation, and poor adherence, hence suboptimal levels of drug and lower CD4/CD8 ratios in ART. The emergence of both the majority and minority drug variants, the type of ART regimen, and the difference in penetration of ART into cellular and tissue reservoirs have also been shown to cause or sustain PLLV [8, 21, 22]. It is therefore important to intensively monitor the adherence and presence of drug-resistant mutants (DRMs) in these patients. Pretreatment factors include high baseline RNA and proviral DNA, nadir CD4+ T-cell counts, preexisting DRMs, and HIV WHO stages [7, 8, 21, 22]. In the present study, 22% of the patients had baseline VL > 1000 copies/ml and 40% had VL 20-999 copies/ml. This could be attributed to the high percentage of patients who experienced PLLV. Unfortunately, DRMs and CD4 data were missing, and therefore, conclusions based on these variables cannot be drawn.

In the study population, 89% of the patients ended up suppressing viremia to below levels of quantification, unlike 11% who transitioned to VF. This could be a result of differences in the level of quality of care by different programs or guidelines by NASCOP in Kenya that recommend similar intensive care among PLLV patients like patients experiencing VF [14]. This could mean there is rigorous treatment and monitoring of the patients, hence the large number of cases transitioning to nondetectable levels. This trend has been previously reported in several countries in Africa including Kenya [5, 23]. Furthermore, Kiweewa et al. [24] reported that a sizeable number of participants with PLLV in their study did not experience VF. The risk of VF did not increase among LLV patients in a Swedish cohort [4]. However, the results of other studies indicated that PLLV was an independent risk factor for VF [9, 25, 26]. In Montreal, Canada, 22.7% of patients with VL 50–199 copies/ml, 24.2% with VL 200–499 copies/ml, and 58.9% with VL 500–999 copies/ml at PLLV experienced VF [10]. Virologic failure among patients with PLLV can occur due to a number of reasons including the treatment with unboosted PI-based regimens, use of NRTI combinations only, emergence of DRMs, and viral loads above 200 copies/ml [1, 14, 15]. These factors should be considered by an AIDS care program when managing patients with PLLV to avoid VF. Fortunately, Kenya has adopted dolutegravir (DTG), an INSTI, to be used as a first-line regimen drug. However, DRMs and viral load monitoring need to be scaled up in patients with PLLV.

The mean age (29 years) at diagnosis in our study was a risk factor for VF. In addition, adolescents, youth, and young adults were at a higher risk of VF than the other age groups. Similar findings were reported in an Austrian cohort where patients under 30 years were more likely to experience VF (OR 2.76; 95% CI, 1.03–7.35) [27]. A large African prospective, multicenter cohort study reported similar results [24]. The VF could be due to nonadherence previously reported among adolescents and young adults in the hospital where this study was done. In a previous study, a high nonsuppression rate was a result of missed clinic appointments (48.3%) and ART refill appointments (50%), which are important factors in evaluating adherence [28]. According to Leierer et al., [27], poor adherence as determined by ART interruptions was associated with VF. Among adolescents and young adults, nonadherence has been previously reported in a study on treatment outcomes among adolescents in South Africa [29], where older age was associated with better adherence in the workplace and community [30]. Teenagers and young adults are often in transition, and as a result, they experience physiological, psychological, and physical changes that influence their behavior in regard to their health [24, 28, 29]. This means that health practitioners need to give special attention to adolescents and young adults as they could hinder the achievement of the 3^rd^ 90 target to achieve suppression.

Results from the present study indicate that patients who had baseline viral loads ≥ 200 copies/ml were more likely to experience VF than those with VL ≤ 199 copies/ml. This could indicate the presence of high HIV-1 quasispecies diversity prior to treatment; hence, patients were not able to suppress viremia following six months of ART (see the baseline viral load definition). In addition, it suggests that patients had very high viral loads prior to treatment. High viral loads (>500000 viral copies/ml) prior to treatment have been reported to delay the efficacy of NNRTI-based ART with incomplete viral suppression [9, 31]. Notably, at the start of PLLV, patients with VL 200-499 copies/ml and those with VL 500-999 copies/ml were three and four times, respectively, more likely to fail compared to those with their first VL measurement between 20 and 199 copies/ml. In addition, those whose VL measurements were all consistently ≥200-999 copies/ml while at PLLV were 14 times more likely to experience VF compared to those with viral blips (five times) and those whose viral load measurements were consistently ≤199 copies/ml. This observation adds to the evidence that VL above 200 copies/ml at PLLV is a risk factor for VF, as reported by previous studies [1, 8, 26, 32, 33]. For instance, an LLV of 200–999 copies/ml was a risk factor for VF (OR 3.14), unlike an LLV of 50–199 copies/ml (OR 1.01) [4]. Patients with high PLLV (500-999 copies/ml) are 2.36 times more likely to fail compared to those with medium PLLV (200-499 copies/ml) and those with undetectable viral loads [5]. Laprise et al. [10] observed an increased risk of VF among patients with increasing viral loads: 22.7% of patients with 50–199 copies/ml (95% CI, 14.9–33.6), 24.2% with 200–499 copies/ml (95% CI, 14.5–38.6), and 58.9% with 500–999 copies/ml (95% CI, 43.1–75.2), compared to 6.6% with an undetectable RNA viral load (95% CI, 5.3–8.2). A study among ART-experienced patients reported that PLLV ≥ 50-500 copies/ml was associated with VF [8]. Boillat-Blanco et al. [1] reported VF of 12% and 22% among patients with PLLV values of 20-199 copies/ml and 200-500 copies/ml, respectively. Virologic failure as a result of unsuppressed VL ≥ 200 copies/ml could be attributed to subtherapeutic drug levels as a result of suboptimal adherence to ART, continued viral replication and selection of HIV-1 DR, NRTI combinations only, and treatment with unboosted PIs among other factors [1, 4, 9, 15]. The present study recommends optimized HIV care to target viral suppression < 200 copies/ml. Results from the present study also reinforce findings from other studies recommending revision of the definition of VF by the World Health Organization for resource-limited countries.

To give information on the management of PLLV, several studies have evaluated the effect of regimen change during PLLV. Some studies have reported a positive impact, that is, suppression [1, 16, 17], while others did not report any significant change [19]. The findings from the present study show that regimen change did not have a significant association with the virologic outcome. However, a change from a first-line to a second-line regimen was associated with a virologic outcome. Patients who changed from a first-line to a second-line regimen were more likely (*P* = 0.028; OR 3.203) to fail virologically compared to those who changed to another first-line regimen. Similar results were reported in a study including different African countries and also in an independent study in a South African cohort [6, 24, 30]. A switch to a second-line regimen is recommended for patients who failed a first-line regimen, as indicated by treatment failure and high viral loads. The probable cause of the high risk of VF among patients who switched to a second-line regimen could be poor adherence, as reported in other studies [6, 24, 30]. Johnston et al. [27] reported that the patient, community, and health system factors were associated with second-line regimen failure. Other factors could include the presence of DRMs. In Kenya, an HIV drug resistance genotyping test is recommended when treatment failure is confirmed on a protease inhibitor-based first-line regimen or failure on a second-line regimen or subsequent regimen [14]. Unfortunately, this is not done consistently due to the prohibitive cost associated with drug resistance testing, especially in RLS. Therefore, patients may have switched regimens without undergoing a drug resistance test (DRT). Although drug resistance was not investigated in the present study, other studies have reported the presence of DRMs among patients with high PLLV (200-999 copies/ml) values [9, 15, 27, 34].

In the present study, a switch to another first-line regimen was inversely associated with VF. The patients who had a start regimen of 2NRTI+1NNRTI significantly changed the regimen compared to other start regimen drug combinations. Regimen change to another first-line regimen could have been a result of Kenya adopting dolutegravir (DTG), an Integrase Strand Transfer Inhibitor (INSTI), as a first-line regimen drug in 2017. Patients who were still taking efavirenz and nevirapine (NNRTIs) have been over time switched to DTG except in a few cases [14]. Dolutegravir is an INSTI that has been reported to be well tolerated, to have a high genetic barrier to HIV drug resistance, and to have high potency and efficacy of viral suppression [5, 35]. This could have contributed to the suppression of nondetectable levels among the patients to whom this drug was introduced in their regimen. In Kenya, by October 2020, 87% of PLHIV had transitioned to DTG-based regimens with notable improvement in suppression overall [35]. The transition from NNRTI-based regimens to DTG-based regimens should therefore be intensified, especially among patients who take earlier first-line regimens without DTG. Regimen change during PLLV, especially for patients with VL > 200 copies/ml, could result in progression to undetectable levels of HIV RNA in the plasma [1, 5, 35, 36]. However, regimen change faces two main challenges. Without sufficient evidence, the dilemma lies in discontinuing patients from taking a drug to which they are not resistant and initiating a drug to which they may be resistant [9]. Secondly, the availability of new drug classes, especially in RLS, may not be guaranteed.

Married marital status was inversely associated with virologic failure (*P* = 0.026) (OR 0.32; 95% CI, 0.12-0.87). Patients who were married were less likely to transition to VF compared to patients with other marital statuses. This was also observed by Kiweewa et al. [22], who reported that single marital status was a predictor for VF while marriage was protective. This could be a result of the psychosocial support from their partners, hence better adherence and other health-related behaviors. Although this study did not investigate psychosocial support, it has been reported to reduce stigma, hence better adherence [37, 38]. Of all the opportunistic infections and noncommunicable diseases evaluated, only a history of oral candidiasis was close to the level of significance (*P* = 0.08) in relation to VF. Patients with a history of oral candidiasis were 2.4 times more likely to fail virologically than their counterparts. Oral candidiasis among people living with HIV/AIDS is an indicator of immune suppression and severity of the disease. Formal education was not related to virologic outcomes. This is encouraging for ART programs and health practitioners serving populations with low levels of formal education. Gender was also not predictive of virologic outcomes among PLLV patients. This was also reported in a 10-year prospective study in a Ugandan cohort [39].

## 5. Conclusion

This retrospective longitudinal study characterized patients with PLLV and recorded baseline viral loads > 200 copies/ml, consistently high viral loads of 200-999 copies/ml at PLLV, viral blips, and high VL > 200 copies/ml at the beginning of PLLV, including adolescents and young adults, which were all significant risk factors for VF that should be monitored during the management of these patients. In addition, the study shows that regimen switch was not a significant factor for virologic outcomes. However, a switch from a first-line to a second-line regimen was a significant risk factor for VF. Monitoring and support need to be intensified among adolescents, young adults, and patients who have switched to second-line regimens. In addition, the management of adolescents and young adults experiencing PLLV needs to be prioritized, as they may present a barrier to progress toward acquiring the third “90” in the 90-90-90 strategy and HIV cure. One of the limitations of this study was that the baseline viral load tests were measured after six months of treatment except for pregnant and breastfeeding mothers and children who were tested before treatment; this disparity could affect the effect of the variable on outcomes. The thresholds of PLLV and VF differ from study to study, making it difficult to compare previous studies. The findings from the present study and evidence from previous studies show that patients in RLS would benefit immensely if WHO adopted the American DHHS guidelines that define VF as persistent viremia ≥ 200 copies/ml rather than lenient 1000 copies/ml. Therefore, in the interim period, AIDS care programs should target to reduce VL to below 200 copies/ml.

## Figures and Tables

**Table 1:** Definitions of terms as used in this study.

Term	Definition
PLLV	Viral loads between ≥20 and 999 copies/ml on at least two consecutive measures
Virologic failure (VF)	Viral loads ≥ 1000 copies/ml
Viral blips	Viral load consistently ≤50 copies/ml with one viral load measurement ≥ 200-999 copies/ml
Nondetectable VL	Viral loads < 20 copies/ml
Baseline VL	The first viral load test Of importance to note is that viral load tests before the commencement of ART are only done on HIV-positive pregnant mothers who test positive while pregnant or who become pregnant when already enrolled for ART care and their children after birth. Other patients who test HIV positive have to take ARVs for six months before their viral load is tested

**Table 2:** Baseline characteristics of patients with PLLV (categorical variables) between 2015 and 2021 at Kenyatta National Hospital.

Variables	Frequency	%
	*N* = 705	
*Age group*		
1 to 9	42	6.0
10 to 19	43	6.1
20 to 24	22	3.1
25 to 30	31	4.4
31 to 40	127	18.0
41 to 50	240	34.0
51 and above	200	28.4
*Age description*		
Child	42	6.0
Adolescent	43	6.1
Youth	22	3.1
Older adults	598	84.8
*Gender*		
Male	317	45.0
Female	388	55.0
*Education*		
None	4	0.6
Preschool	16	2.3
Primary	133	18.9
Secondary	156	22.1
Postsecondary	88	12.5
Not documented	308	43.6
*Marital status*		
Child	63	8.9
Single	163	23.1
Married	358	50.8
Separated/divorced	40	5.7
Widowed	49	7.0
Not documented	32	4.5
*Age at HIV diagnosis*		
1 to 9	74	10.4
10 to 19	18	2.5
20 to 29	57	8.1
30 to 39	133	18.9
40 to 49	71	10.1
50 and above	28	4.0
Not documented	324	46.0
*Years positive*		
0 to 5	58	8.2
6 to 10	140	19.9
11 and above	194	27.5
Not documented	313	44.4
*Baseline WHO stage*		
Stage 1	244	34.6
Stage 2	95	13.5
Stage 3	155	22.0
Stage 4	59	8.4
Not documented	152	21.5
*Baseline viral load*		
Nondetectable	263	37.3
20 to 999	281	39.9
1000 and above	161	22.8
*TB history*		
Yes	106	15.0
No	599	85.0
*OI history*		
Yes	555	78.7
No	150	21.3
*PCP*		
Yes	4	0.6
No	701	99.4
*Cryptococcal disease*		
Yes	4	0.6
No	701	99.4
*Oral candidiasis*		
Yes	32	4.5
No	673	95.5
*Kaposi's sarcoma*		
Yes	3	0.4
No	702	99.6
*Other cancers*		
Yes	15	2.1
No	690	97.9
*Hypertension*		
Yes	92	13.0
No	613	87.0
*Diabetes*		
Yes	23	3.3
No	682	96.7
*Renal disease*		
Yes	22	3.1
No	683	96.9
*IPT status*		
Completed	401	56.9
Deferred	137	19.4
Start	21	3.0
Continuing	35	5.0
Declined	10	1.4
Defaulted	4	0.6
Not documented	97	13.7
*Start VL at PLLV*		
20 to 199	532	75.5
200 to 499	113	16.0
599 to 999	60	8.5
*Start regimen*		
2NRTI+1PI/r	133	18.9
2NRTI+1NNRTI	545	77.3
2NRTI+1INSTI	27	3.8
*Switched regimen at PLLV*		
Yes	329	46.7
No	376	53.3
*Baseline CD4*		
<500	103	14.6
≥500	28	4.0
Not documented	574	81.4

**Table 3:** Comparison of patients who resulted in nondetectable viral loads (<20 copies/ml) and virologic failure (≥1000 copies/ml) after PLLV.

Variables	Nondetectable	VF	*P* value
*N* = 506	*n* = 451	*n* = 55	
*Age group*			
1 to 9	33	4	**0.006**
10 to 19	30	8	
20 to 24	10	4	
25 to 30	11	5	
31 to 40	73	6	
41 to 50	164	17	
51 and above	130	11	
*Gender*			
Male	206	22	0.424
Female	245	33	
*Education*			
None	1	1	0.371
Preschool	13	2	
Primary	91	13	
Secondary	97	11	
Postsecondary	60	5	
Not documented	189	23	
*Marital status*			
Single	93	18	**0.001**
Married	245	18	
Separated/divorced	22	4	
Widowed	26	6	
Not documented	65	9	
*Years positive*			
0 to 5	33	4	0.96
6 to 10	114	12	
11 and above	137	14	
Not documented	222	55	
*Age at HIV diagnosis*			
1 to 9	55	9	**0.002**
10 to 19	10	6	
20 to 29	38	3	
30 to 39	104	6	
40 to 49	55	4	
50 and above	18	2	
Not documented	171	25	
*Baseline CD4*			
<500	74	11	0.525
≥500	11	1	
Not documented	366	43	
*Baseline WHO stage*			
Stage 1	158	20	0.424
Stage 2	65	5	
Stage 3	96	17	
Stage 4	31	5	
Not documented	101	8	
*Baseline viral load*			
Nondetectable	110	8	**0.0001**
20 to 199	213	10	
200 to 999	44	13	
1000 and above	84	24	
*TB history*			
Yes	62	12	0.11
No	389	43	
*OI history*			
Yes	372	46	0.831
No	79	9	
*PCP*			
Yes	3	0	0.544
No	448	55	
*Cryptococcal disease*			
Yes	3	1	0.362
No	448	54	
*Oral candidiasis*			
Yes	18	5	0.086
No	433	50	
*Kaposi's sarcoma*			
Yes	1	0	0.727
No	450	55	
*Other cancers*			
Yes	6	1	0.77
No	445	54	
*Hypertension*			
Yes	69	6	0.387
No	382	49	
*Diabetes*			
Yes	16	0	0.156
No	435	55	
*Renal disease*			
Yes	16	3	0.482
No	435	52	
*IPT status*			
Completed	251	33	0.106
Deferred	76	17	
Start	15	1	
Continuing	20	0	
Declined	9	0	
Defaulted	2	1	
Not documented	78	3	
*First VL at PLLV*			
500 to 999	31	12	**0.0001**
200 to 499	63	15	
20 to 199	357	28	
*VL status at PLLV*			
Viral blips	132	28	**0.001**
VL ≥ 200 to 999	19	13	
All VL ≤ 199	300	14	
*Start regimen*			
2NRTI+1PI/r	89	11	0.9333
2NRTI+1NNRTI	350	43	
2NRTI+1INSTI	12	1	
*Switched regimen at PLLV*			
Yes	184	17	0.157
No	267	38	

VF: virologic failure; OI: opportunistic infection; VL: viral load; WHO: World Health Organization; IPT: Isoniazid Preventive Therapy; PCP: pneumocystis pneumonia.

**Table 4:** Correlates of virologic failure among patients who experienced PLLV at Kenyatta National Hospital between 2015 and 2021.

Variables	Frequency	*n* = 55	OR (95% CI)	*P* value
	*N* = 506	*n* (%)		
Age (SD)				**0.043**
Age at HIV diagnosis				**0.002**
Minimum VL at PLLV				**0.007**
Maximum VL at PLLV				**0.0001**
*Age group*				
1 to 9	37	4 (7.3)	1.433 (0.43-4.79)	0.559
10 to 19	38	8 (14.5)	3.152 (1.17-8.51)	**0.024**
20 to 24	14	4 (7.3)	4.727 (1.27-17.57)	**0.02**
25 to 30	16	5 (9)	5.372 (1.58-18.25)	**0.007**
31 to 40	79	6 (11)	0.971 (0.34-2.74)	0.956
41 to 50	181	17 (31)	1.225 (0.56-2.71)	0.616
51 and above	141	11 (20)	1	
*First viral load*				
1000 and above	108	24 (43.6)	3.929 (1.68-9.18)	**0.002**
200 to 999	57	13 (23.6)	4.062 (1.79-10.48)	**0.004**
20 to 199	223	10 (18.3)	0.646 (0.25 -1.68)	0.37
Nondetectable	118	8 (14.5)	1	
*VL status at PLLV*				
Viral blips	160	28 (51)	4.545 (2.32-8.91)	**0.0001**
All VL > 200 to 999	32	13 (23.6)	14.662 (6.05-35.56)	**0.0001**
All VL ≤ 199	314	14 (25.4)	1	
*Start VL at PLLV*				
500 to 999	43	12 (22)	4.935 (2.29-10.65)	**0.0001**
200 to 499	78	15 (27)	3.036 (1.53-6.00)	**0.0001**
20 to 199	385	28 (51)	1	

## Data Availability

The dataset supporting the conclusions of this paper is available upon request from the corresponding author. However, the personal data of the patients are restricted by the Kenyatta National Hospital/University of Nairobi Ethical Review Committee in order to protect patient privacy.
